# The Enigma of West Syndrome: A Case of Infantile Spasms Without Genetic Clues

**DOI:** 10.1155/crpe/8513643

**Published:** 2025-11-29

**Authors:** Ali Raafat Ammar, Alyaa Al Ramah, Jahnavi Bodi, Nuha AlZaabi, Malay Jhancy

**Affiliations:** ^1^Faculty of Medicine, Ras Al Khaimah Medical and Health Science University, Ras Al Khaimah, UAE; ^2^Metabolic Geneticist Consultant, Fujairah Hospital Emirates Health Service, Fujairah, UAE; ^3^Department of Pediatrics, Faculty of Medicine, Ras Al Khaimah Medical and Health Science University, Ras Al Khaimah, UAE

**Keywords:** case report, epileptic encephalopathy, infantile epileptic spasms syndrome (IESS), myoclonic seizures, West syndrome

## Abstract

West syndrome is a distinctive type of epilepsy characterized by infantile spasms, hypsarrhythmia on EEG, and developmental regression. It affects children between 4 months and 2 years old, with an incidence of 2–3.5 per 10,000 births, being more common in boys. The syndrome is classified into symptomatic, idiopathic, and cryptogenic forms, based on underlying causes such as brain trauma, malformations, infections, chromosomal abnormalities (e.g., Tuberous Sclerosis Complex), or genetic mutations in genes like ARX and CDKL5. This article presents a case of West syndrome in an 8-months-old female without a known genetic cause, despite extensive genetic analysis. The patient presented to us with unprovoked spasms occurring over 2 months. Evaluation through EEG confirmed modified hypsarrhythmia, and MRI revealed significant brain lesions. A comprehensive metabolic panel was negative, and whole exome sequencing for the patient and her parents was inconclusive. This finding is not uncommon, as an estimated 40% of West syndrome cases remain without a confirmed genetic diagnosis. Current treatment recommendations by pediatric neurologists in the GCC include first-line therapy with vigabatrin alone or in combination with high-dose oral corticosteroids. If no response occurs within 2 weeks, ACTH or corticosteroids are added. ACTH, though effective, is less favored due to accessibility, cost, and relapse rates. Vigabatrin has the fewest side effects, followed by corticosteroids, with ACTH presenting the most, particularly irritability and hypertension. This case highlights the challenges in diagnosing and managing West syndrome, especially with inconclusive genetic analysis. It emphasizes the need for continued advancements in genetic diagnostics to uncover new mutations and improve patient outcomes.

## 1. Introduction

West syndrome (WS) is a distinct type of epilepsy marked by a triad of symptoms: infantile spasms, sudden forward bending of the body accompanied by stiffening of the limbs, often occurring in clusters upon waking, hypsarrhythmia on an electroencephalogram (EEG), and a halt in developmental progress. It typically affects children between 4 months and 2 years old but can persist beyond that age range. Around 70%–75% of cases have an identifiable cause, which can include brain trauma, malformations, infections, chromosomal abnormalities like Tuberous Sclerosis Complex (TSC), or mutations in genes like Aristaless-related homeobox (ARX) or cyclin-dependent kinase-like 5 (CDKL5). Incidence ranges from 2 to 3.5 per 10,000 births, with a higher prevalence in boys. WS is categorized into symptomatic, idiopathic, and cryptogenic forms based on underlying causes, which also influence prognosis [1].

In this article, we present a case of WS in an infant who underwent extensive genetic evaluation without conclusive results, highlighting ongoing diagnostic challenges in this field.

## 2. Case Presentation

An 8-months-old female infant, born to nonconsanguineous parents, was brought to Fujairah Hospital in the United Arab Emirates (UAE) with a chief complaint of unprovoked spasms persisting over the past 2 months. As reported by the mother, these spasms occur suddenly, happening approximately 3–4 times a day. A recorded video of the episodes showed abnormal and sudden full-body flexion contractions resembling “salaam” movements, indicative of infantile spasms.

### 2.1. Birth and Developmental History

The patient was born full-term via normal vaginal delivery, weighing 2.7 kg, without the need for admission to the Neonatal Intensive Care Unit (NICU), and with a satisfactory APGAR score of 8. Prenatal history was unremarkable, with all maternal tests returning normal results. Developmental milestones have been met appropriately, and vaccinations up to her current age of 8 months have been administered on time. She has been consuming mashed food alongside breast milk nutritionally, transitioning from exclusive breastfeeding at 6 months of age. There are no significant past medical issues, and there is no family history of seizures or infantile spasms; the patient's older sibling is in good health. A review of the systems revealed no notable abnormalities.

### 2.2. Clinical Examination

On general examination at 8-months-old, the baby was well-nourished with no pallor, cyanosis, or icterus. Her vital signs were normal, with a height of 71 cm (75th percentile) and a weight of 7.5 kg (50th percentile). No spasm episodes were noted.

A neurological examination at 8 months revealed intact cranial nerves, normal deep tendon reflexes, and age-appropriate muscle strength and tone. However, at the 14-month follow-up visit, the patient exhibited gross hypotonia, with power of 4/5, and had not achieved the expected gross motor milestone of sitting independently, indicating a gross motor developmental delay. No neurocutaneous manifestations were observed, helping to rule out tuberous sclerosis. A summary of clinical milestones is shown in Table 1.

### 2.3. Diagnostic Investigations

**Figure 1:**
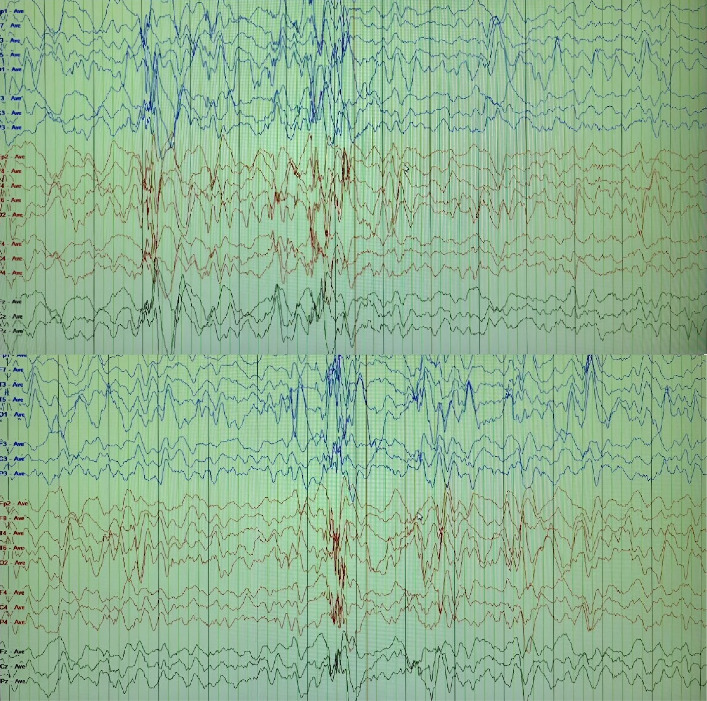
Electroencephalogram demonstrates modified hypsarrhythmia pattern characteristic of West syndrome, showing high-voltage irregular slow waves and multifocal spikes across all cortical regions.

Based on the mother's reports of abnormal movements and video recordings, an EEG was performed and showed modified hypsarrhythmia, initially consistent with infantile spasms (Figure 1). However, the developmental delays observed make the diagnosis more consistent with WS.

### 2.4. Neuroimaging

**Figure 2:**
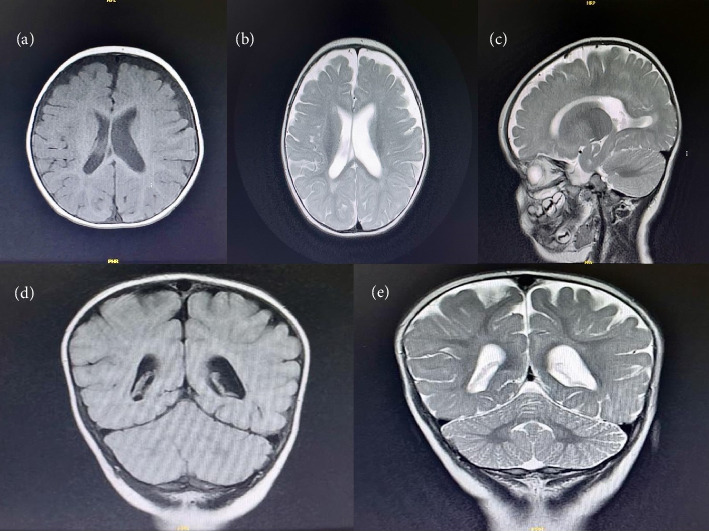
Brain MRI findings: (a and b) Axial T1- and T2-weighted images demonstrate reduced white matter volume along the bilateral lateral ventricles, with more pronounced changes at the trigones. (c) Sagittal T2-weighted image reveals prominent cortical sulci and enlarged extra-axial cerebrospinal fluid spaces, suggestive of cerebral atrophy. (d and e) Coronal T2 FLAIR and Coronal T2 images show hyperintense signal changes, indicating underlying white matter abnormalities.

Brain MRI findings demonstrated reduced white matter at bilateral lateral ventricle trigones with T2/FLAIR hyperintensity, as well as prominent cortical sulci in the fronto-parietal region, sylvian fissures, and extra-axial cerebrospinal fluid (CSF) spaces (Figure 2).

*Metabolic Screening*: A comprehensive metabolic panel was ordered but returned negative results. The results of the metabolic panel are presented in Table 2.

### 2.5. Genetic Analysis

Whole exome sequencing was performed on the patient, and both parents. Unfortunately, the results were inconclusive regarding the genetic study of the patient. Moreover, an epilepsy gene panel has been analyzed and reported as normal.

### 2.6. Treatment and Outcome

Initial treatment included prednisolone at a high dose, which was ordered for the infantile spasms: 40 mg daily for 2 weeks, followed by tapering the dose by 10 mg every 5 days till stopping the dose within 4 weeks.

Nonetheless, the spasm episodes remained uncontrolled, leading the neurologist to switch to zonisamide 25 mg and clobazam 10 mg, which helped manage the spasms.

A comprehensive follow-up plan, including periodic developmental assessments, neurological evaluations, and ancillary investigations, was established to guide the patient's ongoing management.

The mother was counseled on all aspects of care, which include treatment options, risks and benefits, prognosis, management, and current condition.

## 3. Discussion

WS is a type of infantile epileptic spasms syndrome (IESS), an epileptic disorder of infancy and early childhood. It is called WS when 3 major criteria are met: Infantile spasms, psychomotor developmental arrest, and finally an EEG recording produced by electroencephalography showing the abnormality known as hypsarrhythmia. It has an incidence rate of 2–3.5 per 10,000 births and typically begins in the first year of life, as seen in our case. Moreover, it is divided into symptomatic (when a disorder already affects the brain) or cryptogenic (when no previous illness is present) according to the International League Against Epilepsy (ILAE) [2].

As for our case, since all the diagnostic tests and genetic analysis were inconclusive, the diagnosis points towards cryptogenic etiology.

There is very little information available about IESS in the UAE. A team from a pediatric neurology clinic in the UAE has noted that studies on infantile spasms in the region are scarce. They highlighted that cultural and demographic factors complicate the management of IESS. Additionally, no specific publications address WS in the UAE [3].

The diagnosis of WS typically begins when a caregiver notices abnormal symmetric spasms, which may occur in clusters and involve flexor, extensor, or mixed axial jerks. The initial diagnostic step is usually an EEG to confirm the condition. The EEG reveals hypsarrhythmia, characterized by high-voltage random slow waves and spikes across all cortical areas. A 24 h video EEG is the recommended method for this assessment [4].

Following the EEG, an MRI study is necessary to detect any brain pathologies such as cerebral malformations, atrophy, tumors, and other lesions. Additionally, MRI can provide prognostic information; for instance, patients with lesions on their MRI tend to have worse outcomes compared to those with normal MRI findings. As for our case, the patient's MRI showed reduced white matter at the trigons of both lateral ventricles [5].

Additionally, a basic metabolic panel is essential to rule out common metabolic abnormalities in the UAE, such as phenylketonuria, pyridoxine deficiency, and biotinidase deficiency. Finally, whole-exome sequencing can be utilized to identify the underlying genetic cause [6].

Regarding etiology, genetic analysis has identified mutations in over 30 genes, with two novel genes recently discovered. These novel genes, ARX and CDKL5/STK9, have been linked to cryptogenic WS. Both genes are in the Xp22 region of the human chromosome and play a role in fetal development.

Additionally, mutations in other genes, though rare, have been associated with the disease. Examples include mutations in the SLC25A22 and STXBP1 genes [7].

In our case, whole exome sequencing was performed, and the results were inconclusive. This is very rare but according to an article, approximately 40% of cases are of an unknown etiology. Nonetheless, new gene mutations are being discovered with continued advancements in genetic diagnostic technologies [8].

Currently, pediatric neurologists in the GCC recommend the following treatment for IESS. The first-line treatment involves combined therapy with vigabatrin and high-dose oral corticosteroids or vigabatrin alone, followed by an assessment of the clinical response after 2 weeks. If there is no response after 2 weeks, ACTH or corticosteroids are added to vigabatrin. For cases of IESS due to tuberous sclerosis, vigabatrin is used as the first-line treatment [9].

The prednisolone regimen starts with 10 mg four times daily for 14 days, then reduces to 10 mg three times daily from days 15–19, followed by 10 mg twice daily from days 20–24, and finally 10 mg once daily from day 25–29 [9].

ACTH is not recommended as a first-line treatment due to challenges with accessibility, cost, and a high relapse rate after stopping treatment. However, it remains effective for WS. The optimal dose of ACTH is unclear, but a dose of 2–3 IU/kg/day shows a better response than 1–1.9 IU/kg/day and a similar response to 3.1–4 IU/kg/day [9, 10].

Regarding side effects, vigabatrin has the fewest side effects, followed by oral corticosteroids. Intramuscular ACTH has the highest rate of side effects, with irritability and hypertension being the most reported [9].

WS often results in poor long-term outcomes, including a small but significant mortality rate, persistent infantile spasms, other seizure types, and cognitive and psychosocial impairments. Prognosis is primarily influenced by the underlying cause and any pre-existing seizures or developmental issues. Early diagnosis and treatment might affect outcomes, though this remains debated. Families of WS patients also face significant psychosocial challenges, especially if seizures persist beyond early childhood. The natural progression of untreated WS is still unknown, complicating prognosis predictions [11].

For future directions, when comprehensive genetic analysis fails to yield conclusive results, which can be particularly challenging for clinicians, several strategies can be used for further evaluation. Recent studies are starting to emphasize the importance of periodic re-analysis of exome data, as new disease-gene associations are continuously being discovered and variant interpretation improves with expanding genetic databases. This approach has proven valuable, with studies demonstrating that reanalysis can yield additional diagnoses in a proportion of previously unsolved cases [12, 13]. Moreover, emerging technologies such as optical genome has proven successful in identifying complex structural variants that are often missed by traditional sequencing methods. These tools may help uncover novel genes, especially in patients with neurodevelopmental delay and epileptic encephalopathy [14]. RNA sequencing can also provide insights into transcriptional abnormalities not detected by standard genetic testing [15]. Finally, enrolling patients in research initiatives such as the Rare Genomes Project offers access to advanced genomic tools and promotes data sharing, potentially leading to a diagnosis and contributing to broader scientific understanding [16].

## 4. Conclusion

The presented case of an 8-months-old female with WS with no underlying genetic cause, despite extensive genetic analysis, underscores the complexities and challenges in diagnosing and managing this condition. With a significant percentage of cases yielding inconclusive genetic results, there is a critical need for further research. This case highlights the importance of advancing genetic diagnostic technologies to uncover new mutations that could improve patient outcomes.

Moreover, studies regarding WS and IESS are scarce in the UAE despite the presence of unique factors in the region that can complicate the management of these conditions. Therefore, more research is required to help understand the underlying causes and identify the optimal management strategies for WS in the UAE.

## Figures and Tables

**Table 1:** Timeline of key clinical events.

Age	Key event	Clinical finding
Birth	Born full-term	Normal delivery, APGAR 8, no complications
0–6 months	Normal development	All developmental milestones met appropriately
6 months	Spasms begin	First abnormal movements noticed by parents
8 months	Hospital presentation	Video-documented spasms, diagnostic workup initiated
8 months	Diagnosis	EEG confirmed modified hypsarrhythmia
8.5 months	Treatment response assessment	Persistent seizures despite initial therapy
9 months	Seizure control	Spasms controlled with new regimen
14 months	Follow-up	Developmental regression evident, gross hypotonia

**Table 2:** Metabolic panel results.

Test name	Results
Amino acid quantitative analysis of CSF, plasma, and urine	Negative
Ammonia level	Negative
Carnitine-free and total	Negative
Creatine kinase	Negative
Total homocysteine level	Negative
Urine organic acids screen	Negative
Lysosomal enzymes	Negative

## Data Availability

The data that support the findings of this study are available on request from the corresponding author. The data are not publicly available due to privacy or ethical restrictions.
